# Identifying the Components of Acidosis in Patients With Severe *Plasmodium falciparum* Malaria Using Metabolomics

**DOI:** 10.1093/infdis/jiy727

**Published:** 2018-12-19

**Authors:** Stije J Leopold, Aniruddha Ghose, Erik L Allman, Hugh W F Kingston, Amir Hossain, Asok Kumar Dutta, Katherine Plewes, Kesinee Chotivanich, Nicholas P J Day, Joel Tarning, Markus Winterberg, Nicholas J White, Manuel Llinás, Arjen M Dondorp

**Affiliations:** 1 Mahidol-Oxford Tropical Medicine Research Unit, Faculty of Tropical Medicine, Mahidol University, Bangkok, Thailand; 2 Centre for Tropical Medicine and Global Health, Nuffield Department of Medicine, University of Oxford, United Kingdom; 3 Department of Internal Medicine, Chittagong Medical College Hospital, Bangladesh; 4 Department of Biochemistry and Molecular Biology and Huck Center for Malaria Research; 5 Department of Chemistry, Pennsylvania State University, State College, PA

**Keywords:** severe, malaria, *Plasmodium falciparum*, metabolic acidosis, metabolomics, gut-barrier integrity

## Abstract

**Background:**

Acidosis in severe *Plasmodium falciparum* malaria is associated with high mortality, yet the pathogenesis remains incompletely understood. The aim of this study was to determine the nature and source of metabolic acids contributing to acidosis in patients with severe falciparum malaria.

**Methods:**

A prospective observational study was conducted to characterize circulating acids in adults with *P. falciparum* malaria (n = 107) and healthy controls (n = 45) from Bangladesh using high-resolution liquid chromatography–mass spectrometry metabolomics. Additional in vitro *P. falciparum* culture studies were performed to determine if parasites release the acids detected in plasma from patients with severe malaria acidosis.

**Results:**

We identified previously unmeasured plasma acids strongly associated with acidosis in severe malaria. Metabolomic analysis of *P. falciparum* parasites in vitro showed no evidence that these acids are released by the parasite during its life cycle. Instead, 10 of the plasma acids could be mapped to a gut microbial origin. Patients with malaria had low L-citrulline levels, a plasma marker indicating reduced gut barrier integrity. Longitudinal data showed the clearance of these newly identified acids was delayed in fatal cases.

**Conclusions:**

These data suggest that a compromise in intestinal barrier function may contribute significantly to the pathogenesis of life-threatening acidosis in severe falciparum malaria.

**Clinical Trials Registration:**

NCT02451904.

## Teaser

Acidosis in severe falciparum malaria is associated with an accumulation of unidentified acids in blood plasma. Clinical and in vitro metabolomic studies identify a potential role for translocation of bacterial acids from the gut into the bloodstream.

Severe malaria remains an important cause of premature death in malaria-endemic countries with 435 000 deaths ascribed to malaria infections in 2017, mostly in sub-Saharan Africa [1]. The vast burden of severe malaria is caused by the *Plasmodium falciparum* parasite. Early diagnosis and treatment with parenteral artesunate reduces mortality, but case fatality rates still range between 10% and 30% depending on the extent of vital organ dysfunction [2]. Adjunctive therapies might close this case fatality gap if they target pivotal pathophysiological pathways, but this requires a further understanding of the pathophysiology of severe malaria. To date, the only adjunctive interventions shown to reduce severe malaria mortality are renal replacement therapies in acute renal failure. All others have failed.

Unrestrained asexual replication of *P. falciparum* during the blood stage of malaria infection leads to coma, renal failure, and metabolic acidosis [3]. The key pathogenic mechanism in severe falciparum malaria is sequestration of infected red blood cells in the microcirculation of vital organs [4]. This is mediated by parasite-derived ligands, mainly *Pf*EMP1, and a range of host endothelial cell surface receptors, including CD36, EPCR, and ICAM1 [5]. Microvascular sequestration contributes to endothelial dysfunction, capillary and venular blockage, and subsequent organ failure [6]. Histopathology studies have shown preferential sequestration of infected erythrocytes in the brain and intestines [7, 8].

Metabolic acidosis is one of the strongest predictors of mortality in both pediatric and adult cases of severe malaria [6, 9–11]. Acidosis is harmful in itself as it dysregulates cellular metabolism [12]. In severe malaria, acidosis is also a marker of tissue dysoxia, mainly caused by anaerobic production of L-lactate [13]. Depending on the malaria parasite biomass, parasite-derived anaerobic metabolism of glucose to lactate also contributes but is thought to be a minor contributor to the total lactic acid load [14]. L-lactate is the major known acid contributing to acidosis, but does not account fully for the degree of acidosis observed [10, 15]. The remaining unexplained acids in severe malaria are of independent prognostic importance, but they have not been well characterized and their source is not known.

In this study we used a metabolomics screening method to characterize the contributors to metabolic acidosis in patients with severe falciparum malaria and to identify their potential sources. Secondary objectives were to explore if acids found in the plasma of acidotic patients are produced by *P. falciparum* in vitro, and to investigate their clearance kinetics in patients with severe malaria.

## METHODS

### Ethical Considerations

The clinical study protocol was reviewed and approved by the local ethics review board; the Chittagong Medical College Ethics Committee, Bangladesh; and the Oxford Tropical Research Ethics Committee, United Kingdom. The trial was registered at ClinicalTrials.gov (identifier NCT02451904). All participants provided informed written consent after the nature and possible consequences of the studies were explained. If patients were clinically too unwell to provide consent themselves, their attending relatives provided informed written consent.

### Study Design

A prospective observational study was conducted in Chittagong Medical College Hospital, Bangladesh. Chittagong Medical College Hospital is a large government tertiary referral hospital with approximately 1300 beds, serving an urban population of approximately 5 million people. At the time of the study, the hospital had 3 internal medicine wards with a bed occupancy rate regularly reaching >150%. Hemodialysis was available in a separate renal unit operating 8 hemofiltration machines. The hospital has an intensive care unit with 12 beds of which 7 were equipped with mechanical ventilators at the time of the study.

Male and female adults (>12 years) with a blood slide positive for asexual blood stages of *P. falciparum* (including mixed infection with nonfalciparum species) were recruited between 2014 and 2015. Severe and uncomplicated falciparum malaria was defined according the World Health Organization (WHO) criteria for severe malaria [16], modified by Hien et al [17]. Severe acidosis was defined according to WHO criteria [16]. Acute kidney injury was defined according to the Kidney Disease: Improving Global Outcomes (KDIGO) criteria (www.kdigo.org).

At enrollment we took a medical history, assessed vital signs, performed a physical examination, and drew blood samples for blood gas analysis, measurement of *Pf*HRP2, and metabolomic analysis of plasma. During hospitalization, we performed follow-up every 6 hours with vital signs to assess clinical recovery and blood sampling to assess asexual parasite clearance. Patients were treated with standard doses of intravenous artesunate followed by a full course of artemether-lumefantrine [16] and were followed until hospital discharge.

### Sample Size

The effect size of individual metabolites between study groups was unknown a priori, and no pilot studies had been done before. Consecutive patients with malaria were enrolled. We aimed to include patients with different levels of disease severity to observe a wide range of natural variation in the degree of acidosis.

### Plasma Samples for Metabolomics

Blood for metabolomic analysis was drawn from a fresh in-dwelling catheter in the forearm, collected in lithium-heparin tubes, centrifuged immediately, and flash-frozen in liquid nitrogen. Serial plasma samples were extracted in duplicate using a solid-phase extraction technique [18] and by protein precipitation using methanol [19]. Analysis sequences were prepared by regularly interspersing quality control samples (1:10) for post hoc quality control robust locally estimated scatterplot smoothing (LOESS) signal correction [20].

### *Plasmodium falciparum* 3D7 Cultures

*Plasmodium falciparum* 3D7 (*Pf*3D7) strain parasites were cultured and maintained using standard methods [21]. The cultures were synchronized twice using 5% sorbitol using standard procedures with an interval of 6 hours immediately before media sampling. Cultures maintained at 2% hematocrit were split across 3 flasks to achieve parasitemias of 1%, 2%, and 3%. An uninfected red cell control was prepared at 2% hematocrit with blood from the same donor. Media in all flasks were replenished 1 hour prior to the first sampling time point, and were not refreshed afterwards. Duplicate media samples were collected from all flasks at 5 time points across the life cycle, to include a small ring stage (12 hours), large ring stage (20 hours), trophozoite stage (32 hours), schizont stage (40 hours), and schizogony stage (52 hours). At each time point, parasitemia was assessed by microscopy, and cell counts were done using a hemocytometer.

### Mass Spectrometry Measurement, Spectral Data Processing, and Annotation

Ultra-high performance liquid chromatography Orbitrap mass spectrometry (LC-MS) was performed using a C18 column (Hydro-RP, Phenomenex) with tributylamine as an ion-pairing agent, analyzed over a 25-minute run-time protocol in negative ion mode, covering an m/z range of 70–1000 Da on a Thermo Exactive Plus Orbitrap (ThermoFisher) [22] (Supplementary Materials). Base peak chromatograms are shown in Supplementary Figures 2–4. Following peak selection and rigorous quality control, peak annotation was carried out using an in-house library of 292 chemically confirmed metabolic intermediates from major human and *P. falciparum* metabolic pathways. Unidentified peaks were putatively annotated using Human Metabolome Database (HMDB; version 4.0) datasets [23] with confidence at an m/z tolerance of 10 ppm. We filtered the final dataset down to only include acids (based on selection of pKa < 7.0). The metabolomic data analysis pipeline is shown in a flow diagram (Supplementary Figure 1).

### Measures of Intestinal Integrity

We performed an independent LC-MS assay to quantify the plasma levels of L-citrulline and L-arginine. L-citrulline is a nonproteinogenic amino acid produced in enterocytes (predominantly) from plasma glutamine and it is an important substrate for plasma L-arginine [24]. Loss of enterocyte mass reduces splanchnic L-citrulline metabolism [25]. Lowered plasma L-citrulline has been associated with increased gut permeability and endotoxemia [26].

### Statistical Analysis

Data were explored first by principal components analysis (PCA) and by comparing the log_2_ (fold change) between study groups. Welch *t* tests with Benjamini–Hochberg corrections were used for calculation of *P* values for individual metabolites, based on the assumption of unequal variance. Univariate regression analysis of standard base deficit (corrected for albumin) and plasma organic acids was done using a significance analysis of microarrays (SAM) regression analysis tool (‘samr’ package, R) which allowed the calculation of local false detection rate (FDR) and *q* value [27]. Metabolites were selected if regression showed an FDR ≤ .15 and *q* ≤ .001. Log_2_ (fold change) was calculated for individual metabolites and visualized in a heat map for patients with severe malaria, stratified by the degree of acidosis. Spearman correlation coefficients were calculated for assessing correlations. Mann–Whitney *U* and Kruskal–Wallis tests were used for comparing nonnormally distributed variables.

## RESULTS

In total, 152 patients were recruited in this study: 60 with severe malaria of whom 20 (33%) died, 47 with uncomplicated falciparum malaria, and 45 healthy local controls. Baseline characteristics are shown in Table 1. There were no significant differences between the study groups in age (*P* = .83, Kruskal–Wallis test) or sex (*P* = .67, Kruskal–Wallis test); the majority of the study participants were young males (Table 1).

**Table 1.** Baseline Characteristics in the Study Groups

	Severe Malaria		Uncomplicated Malaria		Healthy Controls			
Characteristic	(n = 60)		(n = 47)			(n = 45)		*P* Value
Age, y	28	(22–40)	30	(22–45)	29	(23–35)	.831
Female sex, No. (%)	21	(35)	13	(28)	16	(36)	.6543
BMI, kg/m^2^	21	(20–24)	22	(19–24)	24	(21–27)	.001
Mortality, No (%)	20	(33)	0		0		< .001
Clinical examination							
Temperature, °C	38	(37–39)	37	(37–38)	36	(36–37)	< .001
Coma depth^a^	10	(8–14)	15	(15–15)	15	(15–15)	< .001
Pulse rate, per minute	110	(99–130)	98	(89–112)	83	(78–91)	< .001
Respiratory rate, per minute	36	(28–44)	24	(23–28)	18	(16–20)	< .001
Mean arterial pressure, mm Hg	82	(72–88)	79	(73–87)	93	(87–100)	< .001
Laboratory assessments							
Parasitemia, parasites/µL^b^	44 868	(24 406–82 483)	3840	(1723–8558)	0	…	< .001
*Pf*HPR2, ng/dL^b^	4356	(3218–5896)	647	(429–977)	0	…	< .001
Glucose, mmol/L	6.7	(5–8.7)	6.7	(5.8–7.8)	5.9	(5.2–6.7)	.216
Sodium, mmol/L	135	(131–138)	134	(132–137)	140	(139–141)	< .001
Potassium, mmol/L	4	(3.6–4.3)	3.4	(3.2–3.7)	3.6	(3.5–3.8)	< .001
Chloride, mmol/L	105	(101–108)	104	(99–107)	103	(102–106)	.153
Phosphate, mEq/L	3.2	(2.5–4.3)	2.7	(2.1–3.4)	3.7	(3.3–4.2)	< .001
Magnesium, mmol/L	2.7	(2.3–3.2)	2.3	(2.1–2.5)	2.8	(2.6–2.9)	< .001
Calcium (ionized), mmol/L	0.9	(0.9–1)	1	(0.9–1.1)	1.1	(1–1.2)	< .001
Creatinine, µmol/L	132	(86–361)	92	(69–102)	71	(57–78)	< .001
Blood urea nitrogen, mmol/L	41	(26–86)	16	(11–25)	7	(5–10)	< .001
Albumin, g/L	27	(24–30)	30	(26–35)	45	(43–49)	< .001
Hemoglobin, g/L	90	(64–109)	97	(80–110)	135	(120–149)	< .001
White blood cells, ×10^3^/µL	9.3	(7–15.3)	6.1	(5.2–7.9)	7.9	(6.8–9.1)	< .001
Platelets, ×10^3^/µL	27	(17–43)	46	(37–95)	233.5	(178.5–277)	< .001
pH	7.38	(7.33–7.43)	7.43	(7.4–7.46)	7.37	(7.35–7.39)	< .001
PaCO_2_, mm Hg	29	(25–34)	34	(31–36)	46	(43–52)	< .001
HCO_3_^–^, mmol/L	17.8	(14.7–19.8)	21.3	(19.1–23.9)	25.6	(24.2–27.6)	< .001
Plasma SBDc, mmol/L^c^	6.6	(4.5–9.7)	2.5	(0.2–5)	–1	(–2.9 to 0.3)	< .001
SIDa, mEq/L	41	(38.2–44.3)	40.2	(38.9–42.3)	46.1	(43–48.3)	< .001
SIDe, mEq/L	25	(22.4–27)	28.7	(25.8–30.5)	35.1	(33–36.5)	< .001
Strong ion gap, mEq/L	15.8	(13.3–19.5)	12.1	(10.8–14.3)	10.4	(8.6–12)	< .001
Anion gap^a^, mmol/L^c^	12.3	(10.3–14.6)	9.3	(6.9–11.2)	7	(5.6–8.4)	< .001
L-lactate, mmol/L	3.6	(2.2–4.9)	1.6	(1.2–1.8)	1.2	(1–1.4)	< .001

Data are presented as median (interquartile range) unless otherwise indicated.

Abbreviations: BMI, body mass index; HCO_3_^–^, bicarbonate, PaCO_2_, partial pressure of carbon dioxide; *Pf*HRP2, plasma *Plasmodium falciparum* histidine-rich protein 2; SBDc, standard base deficit; SIDa, apparent strong ion difference; SIDe, effective strong ion difference.

^a^Depth of coma was assessed by the Glasgow Coma Scale (3–15).

^b^Geometric mean (95% geometric confidence interval).

^c^Corrected for plasma albumin and phosphate concentrations.

### Acidosis in Severe Malaria Is Associated With Unmeasured Anions

Acid-base status was assessed by blood gas analysis and estimation of the unmeasured acid load in all participants at enrollment (Supplementary Methods). The majority of patients with severe falciparum malaria had metabolic acidosis. Severe acidosis, defined as a standard base deficit corrected for albumin (SBDc) >8 mmol/L [16], was present in 23 of 60 (38%) severe malaria cases, and moderate acidosis (SBDc >3.3 and ≤8 mmol/L) was found in 28 (47%) cases. Only 9 (15%) severe malaria cases had mild or no acidosis, defined as a standard base deficit corrected for albumin SBDc ≤3.3 mmol/L [28].

**Figure 1.**
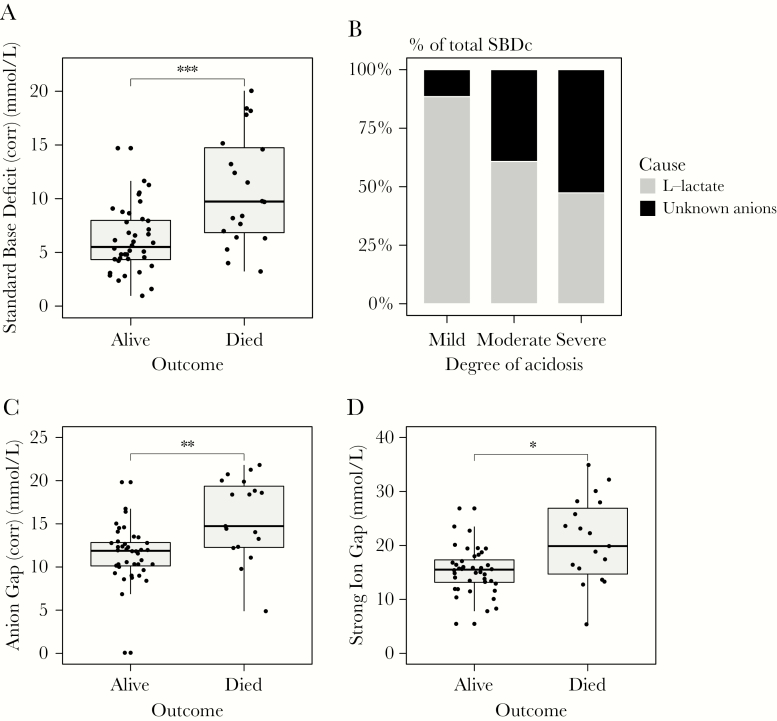
Elevated levels of previously unidentified organic acids in patients with severe *Plasmodium falciparum* malaria. *A*, Standard base deficit corrected for albumin and phosphate (SBDc) in relation to outcome in patients with severe *P. falciparum* malaria (n = 60). *B*, Mean proportion of standard base deficit attributable to L-lactate among patients with mild, moderate, and severe acidosis. *C*, Anion gap corrected for albumin and phosphate. *D*, Strong ion gap. Box plots show median (interquartile range) and minimum and maximum; significance tests were done using Kruskal–Wallis tests. *P < .05, **P < .01, ***P < .001.

The case fatality rate among patients with severe malaria increased stepwise with the degree of acidosis, from 10% in mild acidosis, 21% in moderate acidosis, to 56% in severe acidosis. SBDc was elevated significantly in fatal cases (10.9 mmol/L; standard deviation [SD], 5.1) compared with survivors (6.0 mmol/L; SD, 3.3; *P* = .0004) (Figure 1A). The proportion of acidosis explained by L-lactate decreased with incresing severity of acidosis: L-lactate explained on average 89% (SD, 19%) of SBDc in patients with mild or no acidosis, 61% (SD, 28%) in moderate acidosis, and 47% (SD, 24%) in severe acidosis (Figure 1B).

Further evidence for a prognostic unmeasured acid load was found by estimation of the anion gap (AGc) and the strong ion gap (SIG), both of which were elevated significantly in patients who died (Figure 1C and 1D). SBDc and other estimates of unidentified acids were strongly correlated including AGc (Spearman *r* = 0.61; *P* < .0001) and SIG (Spearman *r* = 0.46; *P* < .0001). As expected, there was also a correlation between SBDc and creatinine (Spearman *r* = 0.43; *P* < .0001). Acute kidney injury was present in 19 of 51 (37%) patients with acidosis, so the majority of patients with acidosis did not have evidence of renal impairment.

### High-Resolution Metabolomics Reveals Differentially Abundant Metabolites

To characterize contributing organic acids, we analyzed plasma samples of all 152 participants in duplicate using an ultra-high-performance LC-MS (UHPLC-MS) platform. This analytical platform detected 1355 metabolic features in plasma of study participants in a mass-to-charge ratio (m/z) range of 70–1000 Da. The final data contained 86 acids identified using an in-house chemical reference library; if chemical standards were unavailable, metabolites were putatively annotated using HMDB, version 4.0 (http://www.hmdb.ca/) (Supplementary Figure 5).

Sources of variation between the patient samples were explored by PCA, which showed a clear separation according to study group on PC1 (Supplementary Figure 6*A*). Differences between study groups were investigated by differential metabolite abundance analysis, and 23 metabolites were found to be significantly elevated in patients with uncomplicated malaria when compared to healthy controls (log_2_ [fold change] >1, Benjamini–Hochberg adjusted *P* < .01) (Supplementary Figure 6*B*). Furthermore, a total of 26 metabolites were significantly increased in patients with severe malaria compared to uncomplicated malaria (log_2_ [fold change] >1, Benjamini–Hochberg adjusted *P* < .01) (Supplementary Figure 6*C*). The results from the differential abundance analyses are described in Supplementary Figure 7.

A major difference was also observed in the levels of acetaminophen glucuronide in the plasma from patients who had malaria compared to healthy controls (Supplementary Figure 7). Acetaminophen glucuronide is a water-soluble acidic metabolite from hepatic glucuronidation of acetaminophen (paracetamol), showing (unsurprisingly) that patients with malaria were taking antipyretics.

### Metabolomic Analysis of Plasma Identifies Previously Unmeasured Acids Potentially of Microbial Origin

**Figure 2.**
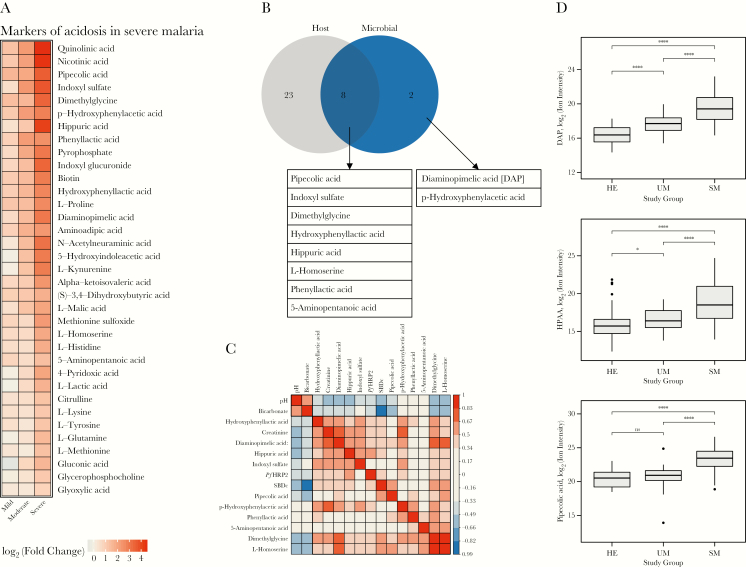
Metabolomic analysis of plasma of patients with severe malaria. *A*, Heat map of all organic acids that were significantly associated with the standard base deficit corrected for albumin and phosphate (false discovery rate < 0.15). Visualization was done by calculating the log_2_ (fold change) normalized against the levels found in patients with uncomplicated malaria, and stratified according to the degree of acidosis. Metabolomic analysis of plasma of patients with severe malaria. *B*, Venn diagram of the biological origin of acids identified in acidotic patients with severe *Plasmodium falciparum* malaria. Acids were mapped to their biological source using the online Human Metabolome Database, version 4.0. *C*, Correlation matrix based on Spearman correlation coefficients of clinical variables of acid-base balance, including blood pH, bicarbonate, base deficit, and 10 microbial acids implicated in acidosis in patients with severe malaria. *D*, Box plots of microbial acids implicated in acidosis. Diaminopemelic acid is a component of gram-negative cell walls; p-hydroxyphenylacetic acid is a product of polyphenol metabolism by gut microbiota; pipecolic acid is a product of lysine metabolism in intestinal bacteria. * = <0.05; **** = <0.0001. Abbreviations: DAP, diaminopemelic acid; HE, healthy controls; HPAA, hydroxyphenylacetic acid; ns, not significant; *Pf*HRP2, plasma *Plasmodium falciparum* histidine-rich protein 2; SBDc, standard base deficit corrected for albumin and phosphate; SM, severe malaria; UM, uncomplicated malaria.

To determine the association between acidosis and previously unidentified organic acids, we performed a SAM regression analysis corrected for multiple testing using FDRs and *q* values among patients with severe malaria. A significant association between SBDc and 36 organic acids was found (FDR < 0.15, *q* < .0001). These organic acids are shown using their log_2_ normalized fold change stratified according to the degree of acidosis (Figure 2A). Significant associations were found between SBDc and metabolites from energy metabolism such as lactate and ketones. There were also increased plasma concentrations of free amino acids found in acidotic patients with severe malaria. There was no relationship between acidosis and drug metabolites from common medications previously linked to the development of acidosis, such as aspirin or acetaminophen [29, 30]. Concentrations of salicyluric acid, an aspirin metabolite previously implicated in the development of acidosis in children with malaria [29], and acetaminophen glucuronide and pyroglutamic acid, acetaminophen metabolites known to cause pyroglutamic acidosis [30], were not elevated.

Significantly associated organic acids were mapped to their metabolic origins using HMDB (version 4.0) data. We found an overrepresentation of organic acids from a microbial origin, and 10 of 36 (28%) metabolites were described as having a microbial source (HMDB, version 4.0) (Figure 2B). These potential microbial acids cross-correlated significantly and were correlated negatively with blood pH and bicarbonate (Figure 2C). Plasma concentrations of these potential microbial acids increased with disease severity (Figure 2D). The 10 organic acids included bacterial cell-wall components and metabolic intermediates (for a review, see Wikoff et al [31] and Nicholson et al [32]; Table 2 and Supplementary Figure 8). Diaminopimelic acid (DAP) concentrations were also elevated in acidotic patients with severe malaria; DAP is a known component of the cell wall of gram-negative intestinal bacteria [33]. A set of 3 phenyl compounds, including p-hydroxyphenylacetic acid, phenyllactic acid, and hydroxyphenyllactic acid, are known intermediates of phenylalanine metabolism in intestinal bacteria [34]. Indoxyl sulfate has been described to derive from bacterial metabolism [31]. The increase in acids potentially derived from an enteric source suggests a role for translocation from the gut, compounded by renal impairment if these acids undergo renal clearance.

**Table 2.** Gut Microbial Acids Implicated in Severe Malaria Acidosis

Name	HMDB ID	Log_2_ (FC)b	pKac	Biological Role/Pathway	Related Bacteria	Reference
Dimethylglycine^a^	HMDB0000092	3.4	1.88	Glycine metabolism	*Bifidobacterium* spp	[32]
Diaminopimelic acid	HMDB0001370	3	…	Gram-negative cell wall component	Gram-negative bacteria	[33]
Hydroxyphenylacetic acid^a^	HMDB00020	2.8	4	Phenylalanine metabolism	*Clostridium* spp *Bifidobacterium* spp *Lactobacillus* spp	[32]
Hydroxyphenyllactic acid^a^	HMDB0000755	2.7	3.58	Phenylalanine metabolism	*Clostridium* spp *Bifidobacterium* spp *Lactobacillus* spp	[35]
Phenyllactic acid^a^	HMDB0000779	2.5	4.02	Phenylalanine metabolism	*Clostridium* spp *Bifidobacterium* spp *Lactobacillus* spp	[36]
5-aminopentanoic acid^a^	HMDB0003355	1.4	4.65	Protein synthesis, amino acid biosynthesis	*Firmicutes* spp	[37]
Pipecolic acid^a^	HMDB0000070	3.6	2.06	Protein synthesis, amino acid biosynthesis	*Escherichia coli*	[38]
Hippuric acid	HMDB0000714	4.1	3.6	Benzoate metabolism	*Clostridium* spp	[31]
Indoxyl sulfate	HMDB0000682	3.7	–1.82	Uremic toxin	*Clostridium sporogenes* *E. coli*	[32]

Gut microbial acids associated with acidosis among patients with severe malaria.

Abbreviations: FC, fold change; HMDB, Human Metabolome Database; pKa, acid dissociation constant.

^a^Identity confirmed by chemical standards, otherwise annotated using HMDB, version 4.0.

^b^Log_2_ (fold change) compared to uncomplicated malaria controls as a clinically relevant reference group.

^c^As stated or predicted in HMDB, version 4.0.

### In Vitro *P. falciparum* Culture Assays Do Not Support a Parasitic Origin of Acids in Severe Malaria

To determine if *P. falciparum* parasite–infected red blood cells release the acids identified in severe malaria acidosis, we performed an in vitro culture study using *P. falciparum* 3D7 strain parasites. A metabolomic analysis was conducted of temporal changes of metabolites in the spent culture media of *Pf*3D7 and nonparasitized red blood cell controls throughout the parasite life cycle. Culture media metabolites were extracted using the same methanol extraction techniques applied to patient plasma, and the samples were analyzed using the same UHPLC-MS platform (Supplementary Figure 4).

**Figure 3.**
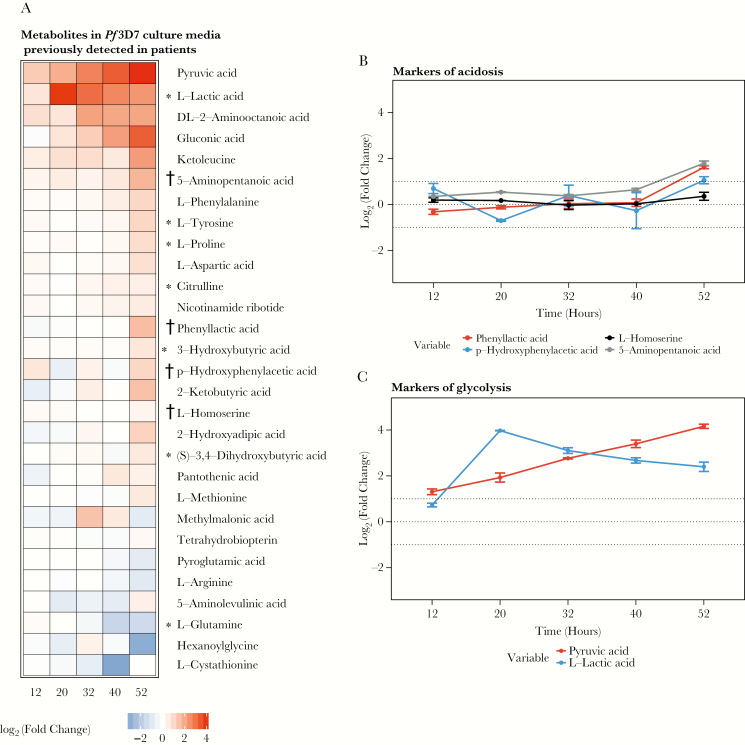
Metabolomic analysis of culture media of *Plasmodium falciparum* 3D7 strain parasites in vitro. *A*, Metabolites secreted by *Pf*3D7 in culture media during the parasite life cycle. The concentration profiles were based on the log_2_ normalized fold change 3% parasitemia cultures, compared to uninfected red blood cell controls. *B*, Secreted microbial markers of acidosis. *C*, Secreted glycolytic metabolites.*Metabolites previously detected in plasma of acidotic patients with malaria.†Metabolites previously identified in plasma of acidotic patients with malaria of a suspected microbial origin.

**Figure 4.**
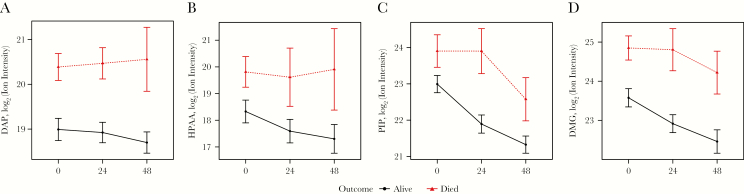
Delayed clearance of suspected microbial markers associated with acidosis. The study hours are displayed on the x-axis. Means and standard errors of the mean are reported of the ion intensity of candidate acids. Serial sampling was done in patients with severe malaria (n = 60) at 0, 24, and 48 hours and temporal dynamics of candidate compounds were compared between survivors (n = 40, black lines) and patients who died (n = 20, red lines). Shown here are 4 of 10 microbial acids identified in the plasma of acidotic patients with severe malaria. *A*, Diaminopimelic acid (DAP). *B*, p-Hydroxyphenylacetic acid (HPAA). *C*, Pipecolic acid (PIP). *D*, Dimethylglycine (DMG).

As expected [35], the release of metabolites from infected red blood cells increased during the trophozoite stage of asexual intraerythrocytic development and peaked after schizogony (Figure 3A). We detected 11 of 36 metabolic acids associated with clinical acidosis in the media of *Pf*3D7 cultures. Except for lactate (parasites produce both D and L forms), none of these acids showed a cumulative increase during the parasite life cycle (Figure 4B). A minor increase was observed following schizogony (52 hours postinfection), indicating the release of red cell content into the media following the rupture of previously parasitized red blood cells (Figure 3B). We did observe an increase in the levels of metabolites related to glycolysis, including pyruvate and lactate (Figure 3C), reflecting the high rates of glycolysis in parasitized red cells [14, 35]. In conclusion, we found no evidence to suggest that *P. falciparum* parasites release any of the microbial acids contributing to acidosis in severe malaria patients.

### Markers Intestinal Barrier Dysfunction in Patients With Malaria

If intestinal leakage is driving the translocation of microbial acids into the circulation, we expected to find markers of reduced gut barrier integrity in patients with malaria compared to healthy controls. To test this hypothesis, we quantified plasma concentrations of L-arginine and L-citrulline and found a significant reduction in L-arginine and L-citrulline levels in patients with *P. falciparum* malaria (*P* < .0001) (Figure 4) [36]. However, reduced intestinal L-citrulline appearance in patients with malaria may also contribute to the low plasma L-arginine levels. We conclude that decreased L-citrulline levels observed in patients with *P. falciparum* malaria are indicative of intestinal barrier dysfunction and are associated with low plasma L-arginine.

### Fatal Cases of Severe Malaria Show Delayed Plasma Clearance of Newly Identified Acids

**Figure 5.**
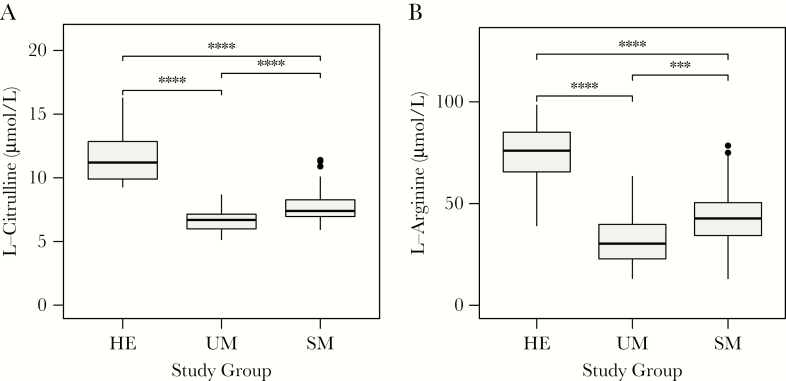
Markers of gut-wall and endothelial function in patients with malaria and healthy controls. Plasma amino acids were quantified using targeted high-performance liquid chromatography–tandem mass spectrometry in plasma of healthy controls (HE), and patients with uncomplicated malaria (UM) and severe malaria (SM). *A*, L-citrulline is a marker of gut wall integrity; reduced blood levels indicate intestinal wall damage. *B*, L-arginine is a marker of endothelial function. **** = <0.0001.

We analyzed the temporal profiles of plasma levels of gut microbial acids during the first 48 hours of hospitalization (Figure 5: the full group of 10 microbial acids is shown in Supplementary Figure 9). All gut microbial acids were elevated in fatal cases and showed a delayed clearance over time in patients with a fatal course, suggesting prognostic relevance. A multivariate logistic regression model including major determinants of outcome in severe malaria showed that besides depth of coma and L-lactate, diaminopimelic acid concentrations were also an independent predictor of outcome (Supplementary Table 1).

## DISCUSSION

In this study, we characterized circulating acids in patients with severe falciparum malaria. LC-MS–based metabolomics revealed elevated levels of acids, which are known to be produced by gut bacteria. Assaying spent medium of *P. falciparum* in vitro cultures at different stages of asexual development did not identify *P. falciparum* as a likely source for these newly identified acids. Patients with malaria had markers indicative of loss of gut barrier dysfunction. Clearance of microbial acids was delayed in fatal cases, supporting their prognostic significance in adult patients with severe malaria. Combined, these findings point toward a potential role for translocation of bacterial acids from the gut into the bloodstream, suggesting a novel pathogenesis for acidosis, contributing to disease severity and poor prognosis in patients with severe *P. falciparum* malaria.

Severe falciparum malaria predisposes to concomitant bacteremia [37], often with enteric bacteria. Increased intestinal permeability in patients with severe malaria has been shown by sugar absorption tests [38, 39]. Endotoxemia, likely arising from translocation of intestinal bacteria or bacterial products, and perhaps compounded by reduced hepatic clearance, has been found in children and adults with severe malaria [40, 41]. The mechanisms underlying intestinal barrier dysfunction in severe malaria are probably related to sequestration of infected red cells in the splanchnic microcirculation [7, 8]. The gut is a favored site for sequestration, and mechanical obstruction in gut capillaries has been visualized in vivo. Reduced gut microcirculatory flow may cause local intestinal ischemia [42]. One proposed model to explain increased intestinal permeability is that of parasite-induced histamine release in the gut wall, leading to loss of enterocyte tight junctions [43]. We found that plasma concentrations of *Pf*HRP2 correlated positively with the newly identified organic acids, suggesting that an increased parasite biomass and subsequent sequestration was associated with translocation of microbial acids (Supplementary Figure 10). Alternative explanations for translocation of microbial acids may include diffuse splanchnic ischemia due to severe hemodynamic shock [44], sepsis [45], or coinfection with invasive enteric pathogens [46]. Further studies are needed to investigate the exact pathogenic mechanisms of increased intestinal permeability during severe malaria. Whether organic acids from an enteric bacterial source also accumulate during other disease states or in critically ill patients with a leaky gut warrants prospective investigation.

In the presence of a compromised intestinal barrier, the composition of the gut microbiota may play a role in the pathogenesis of severe malaria acidosis. Recent mouse studies have found that the severity of malaria was affected by the composition of the gut microbiota [47]. Increased intestinal permeability during malaria infection may coincide with changes in the composition of the gut microbiota [48]; however, this has not been investigated in humans.

Our findings suggest an interaction between the human host, the *P. falciparum* parasite, and the gut microbiota. We postulate that sequestration of parasitized red cells in the splanchnic microcirculation may cause intestinal barrier dysfunction, predisposing patients to translocation of gut microbial acids and enteric bacteria (Supplementary Figure 11). If increased plasma concentrations of bacterial acids signify a loss of gut barrier function, this could be predictive for concomitant gut-derived bacteremia. Unfortunately, quality-controlled blood culture facilities were unavailable at the study site.

It should be noted that although L-citrulline is considered a marker of gut integrity, the observed reduction of L-citrulline in patients with malaria can be compounded by significant scavenging by *P. falciparum* parasites [35]. Additionally, it is possible that long-term storage may also affect the stability of low-molecular-weight molecules, including organic acids and L-citrulline [49]. We do not expect sample degradation as storage time was kept to a minimum and there were clear and significant metabolic differences between patient groups.

*Plasmodium falciparum* clone 3D7 was selected for the in vitro studies because it is very well characterized, grows efficiently in vitro, and has been fully sequenced and well annotated, and the 3D7 intraerythrocytic developmental cycle transcriptome is available [50]. While our analysis is limited to the investigation of this single strain, which might have undergone physiological changes following long-term laboratory adaptation, future studies should seek to include clinical isolates to compare parasite acid production using parasites isolated from patients with or without acidosis. Unfortunately, in the current study, this was not possible.

In conclusion, metabolomic profiling showed elevated plasma concentrations of gut microbial acids during severe malaria acidosis. Our data suggest a novel pathogenic pathway for acidosis in severe malaria, where loss of gut barrier function related to microcirculatory parasite sequestration may cause the translocation of microbial acids into the circulation.

## Supplementary Data

Supplementary materials are available at *The Journal of Infectious Diseases* online. Consisting of data provided by the authors to benefit the reader, the posted materials are not copyedited and are the sole responsibility of the authors, so questions or comments should be addressed to the corresponding author.

Supplementary Material

## Notes

***Author contributions.*** A. M. D., S. J. L., and H. W. K. conceived the study. S. J. L., H. W. K., K. P., A. H., and A. K. D. provided patient care and sample collection. S. J. L., E. L. A., J. T., M. W., and M. L. designed and performed mass spectrometry experiments. S. J. L. and E. L. A. performed data analysis and made the figures. All authors read and approved the final manuscript.

***Acknowledgments.*** We are grateful for all patients and their families who participated in the study. We thank Sanjib Kanti Paul and Sumon Sharma for their support with screening patients; Md Safiqul Mostafa Chowdhury for his administrative support; Marja Schilstra for data management; and Dr Phillip Smith and the Huck Institutes of the Life Sciences–Penn State Metabolomics Core Facility, for the use of mass spectrometry facilities.

***Financial support.*** This work was supported by the Wellcome Trust (grant number 106698/B14/Z); a Nuffield Department of Medicine Prize Studentship of the University of Oxford (to S. J. L.); a Burroughs Wellcome Fund Investigators in Pathogenesis of Infectious Disease award (to M. L.); and a National Institutes of Health Ruth Kirschstein National Research Service Award individual postdoctoral fellowship (F32) (award number AI124507 to E. A.).

***Potential conflicts of interest.*** All authors: No reported conflicts of interest. All authors have submitted the ICMJE Form for Disclosure of Potential Conflicts of Interest. Conflicts that the editors consider relevant to the content of the manuscript have been disclosed.
