# Supplementary material for: Identifying the Components of Acidosis in Patients With Severe Plasmodium falciparum Malaria Using Metabolomics
Source: J Infect Dis. 2018 Dec 19;219(11):1766–76. doi: 10.1093/infdis/jiy727 (PMC6500555; doi:10.1093/infdis/jiy727)
Supplement: Supplementary Material [file jiy727_suppl_supplementary_material.docx]

Supplemental Materials

Identifying the components of acidosis in patients with severe *P. falciparum* malaria using metabolomics

Stije J. Leopold^1,2^, Aniruddha Ghose^3^, Erik L. Allman^4^, Hugh W.F. Kingston^1,2^
Amir Hossain^3^, Asok Kumar Dutta^3^, Katherine Plewes^1,2^, Kesinee Chotivanich^1^,
Nicholas P.J. Day^1,2^, Joel Tarning^1,2^, Markus Winterberg^1,2^, Nicholas J. White^1,2^,
Manuel Llinás^4,5^, and Arjen M. Dondorp^1,2*^,

^1^Mahidol-Oxford Tropical Medicine Research Unit, Faculty of Tropical Medicine, Mahidol University, Bangkok, Thailand,

^2^Centre for Tropical Medicine and Global Health, Nuffield Department of Medicine, University of Oxford, UK

^3^Department of Internal Medicine, Chittagong Medical College Hospital, Chittagong, Bangladesh,

^4^Department of Biochemistry and Molecular Biology and Huck Center for Malaria Research, Penn State University, Pennsylvania, USA,

^5^Department of Chemistry, Penn State University, Pennsylvania, USA,

# Contents

**Supplementary Methods**
**Fig. S1.** Schematic description of metabolic analysis, data normalization and analysis of patient plasma.
**Fig. S2.** Base peak chromatograms of patient plasma extracted using a solid phase extraction (SPE) technique.
**Fig. S3.** Base peak chromatograms of patient plasma extracted using a methanol (MeOH) technique.

**Fig. S4.** **Fig. S4.** Base peak chromatograms of *Pf*3D7 spent media extracted using a methanol protein precipitation (MeOH) technique.
**Fig. S5.** Metabolites detected in plasma collected at study enrolment in study participants (n=152) from Bangladesh.
**Fig. S6. Metabolomic analysis of patient plasma.** (A) Principal component analysis (PCA) showing separation by patient groups. (B) Differential abundance analysis of acids compared between uncomplicated malaria and healthy controls. (C) Differential abundance analysis of acids compared between patients with uncomplicated malaria and severe malaria. The Y-axis denotes -log_10_(Benjamini-Hochberg adj. p-values) and x-axis denotes log_2_(fold change).

**Fig. S7.** Differential abundance analysis of metabolites detected in plasma from patients with uncomplicated malaria (n=47), severe malaria (n=60), and healthy controls (n=45) in a metabolomic analysis (UHPLC-Orbitrap-MS) of plasma.
**Fig. S8.** Chemical characteristics of suspected microbial acids detected in plasma of acidotic patients with severe *P. falciparum* malaria.
**Fig. S9.** Temporal kinetics of suspected microbial acids detected in plasma of acidotic patients with severe *P. falciparum* malaria.

**Fig. S10.** Correlation plots of *Pf*HRP2 with individual organic acids from a likely enteric bacterial source. *Pf*HRP2 is a marker of parasite biomass and indirectly reflect tissue sequestration.

**Figure S11. Proposed mechanism for translocation in severe *P. falciparum* malaria.** Sequestration of parasitized red cells in the splanchnic microcirculation leading to local tissue dysoxia and loss of tight junctions reduces enterocyte integrity, followed by translocation of microbial acids and other bacterial products into the circulation.
**Fig. S12.** Correlation between L-lactate measured using the iStat system and by UHPLC-Orbitrap-MS.

# Supplementary Methods

## Clinical definitions

Severe malaria was defined as any *P. falciparum* parasitemia in adults, detected by asexual stages on a peripheral blood slide or a positive rapid diagnostic test in combination with one or more: (1) GCS $<$11; (2) Hematocrit $<$20% with parasite count $>$100,000/mm; (3) Jaundice with bilirubin $>$2.5 mg/dl with parasite count $>$100,000/ mm; (4) Serum creatinine $>$3 mg/dL (265µmol/L); (5) Hypoglycemia with venous glucose $<$40 mg/dL; (6) Systolic blood pressure $<$80mmHg with cool extremities; (7) Peripheral asexual stage parasitemia $>$10%; (8) Peripheral venous lactate $>$4 mmol/L, (9) Peripheral venous bicarbonate $<$15 mmol/L; (10) Respiratory insufficiency. Full informed written consent was obtained, including fully written informed consent from the relative or parent/guardian in case of reduced consciousness and/or age $<$16 years.

Uncomplicated malaria required the presence of *P. falciparum* asexual stages on a peripheral blood-slide or a positive rapid diagnostic test, and the absence of any of the complications of severe malaria.

Healthy controls were recruited inside the study hospital and usually included family members of hospitalized individuals who were not enrolled in the study. Healthy controls were excluded if they had any chronic health conditions, if they were taking medication, pregnant, or if they had abnormal vital signs (including hypertension). All healthy controls were tested for malaria, they were excluded if any of the malaria tests was positive.

## Clinical measurements and sampling

A clinical history was taken at baseline, including a questionnaire on the history of travel and the administration of medication prior to current admission. Clinical measurements included a record of vital signs and levels of consciousness. A new venous cannula (18G/20G) was placed in the forearm and a single venous blood draw was collected. Samples for venous blood gas analysis and measurements of acid-base balance were collected in blood gas syringes coated with lithium-heparin (Terumo, Tokyo, Japan), mixed, and immediately analyzed using CG4^+^ cartridge for analysis on a portable iStat analyzer (Abbott, Chicago, Illinois). Samples for mass spectrometry analyses were collected in lithium-heparin containers (BD Vacutainer, Reading, United Kingdom), mixed, and immediately transferred to a pre-cooled centrifuge at 4 °C. After centrifugation at 1200rpm for 5 minutes the plasma was transferred into Cryo-vials (Corning, Corning, New York) and flash frozen into liquid nitrogen for on-site storage.

Plasma levels of *Plasmodium falciparum* Histidine Rich Protein 2 (PfHRP2) were measured using a commercially available sandwich ELISA assay (Cellabs, Sydney, Australia), as described previously. [1,2]

## Estimation of unmeasured acids

To estimate the concentration of unmeasured circulating acids, we calculated the standard base deficit, the anion gap and the strong ion gap as described previously.[3] We recorded pH, bicarbonate, sodium, potassium, chloride, and ionized calcium using the iStat system in the same blood sample at the study site. Albumin, phosphate, and magnesium were determined at the Hospital for Tropical Diseases in Bangkok, Thailand, from a serum sample stored at -80 °C.

Standard Base Deficit was calculated using the modified Van Slyke equation[4] and corrected for differences in plasma albumin and phosphate using correction factors developed by Wooten.[5] Here, albumin is expressed in g/dL and phosphate in mg/dL.

SBDc = ([HCO_3_^-^ ] - 24.4 ) + (8.3 * [Albumin] * 0.15 + 0.29 * [Phosphate] * 0.32) * (pH - 7.4)

Furthermore, the anion gap was estimated and corrected for the negative charge of albumin (2.8 mEq/L per g/dL at a pH of 7.4) and phosphate (0.59 mEq/L per mg/dL at a pH of 7.4). Here, albumin is expressed in g/dL and phosphate in mg/dL.

AGc = ([Na^+^ ] + [K^+^ ] ) - ([Cl^-^] + [HCO_3_^-^] ) - (2 * [Albumin] + 0.5 * [Phosphate])

For calculation of the strong ion gap we used Stewart’s Approach[6] modified by Figge et al.[7] and determined the apparent strong ion difference (SIDa) and the effective strong ion difference (SIDe), as follows:

SIDa = ([Na^+^ ] + [K^+^ ] + [Mg^2+^ ] + [Ca^2+^ ] ) - ([Cl^-^] + [Lactate^-^] )

SIDe = (2.46 * 10^^-8^ * pCO_2_ / 10^^-pH^) + ([Albumin] * (0.123 * pH – 0.631) ) + ([PO^4-^ ] * (0.309 * pH – 0.469)))

Finally, the strong ion gap (SIG) was calculated by subtracting the apparent and effective strong ion differences:

SIG = SIDa – SIDe

## Plasma sample preparation

Plasma samples were shipped on dry ice to the Mahidol-Oxford Tropical Medicine Research Unit in Bangkok and stored at -80°C until extraction. A total of 384 samples underwent extraction procedures to recover metabolites. Sample extractions included 1) solid phase extraction (SPE) [8] and 2) protein precipitation using methanol.[9] All samples were randomized and de-identified prior to metabolite extraction procedures and analytical analysis. Aliquots (20µL) were taken from all samples to prepare pooled quality control (QC) samples, fully representative of all patient samples.

Solid phase extraction was performed as described previously.[8] In brief, 100µL of plasma was mixed with an ammonium acetate buffer (pH 8) and spiked with an internal standard solution using a multistepper autopipette. Plates were mixed for 10 minutes at 600 rpm on a MixMate (ThermoFisher, Waltham, Massachusetts) before undergoing solid phase extraction through an Isolute PE-AX (Biotage Uppsala, Sweden) 96-well cartridge according to the manufacturer’s instructions. Extracted metabolites were evaporated to dryness with nitrogen using a TurboVap (Biotage Uppsala, Sweden) at 37 °C for 1 hour prior to storage at -80°C. Protein precipitation was done by adding HPLC grade methanol (FLUKE) (Sigma, St Louis, Missouri) in a 1:10 ratio to 100 µL of plasma using a multi-stepper pipette.[9] Samples were individually vortexed at full speed for 30 seconds followed by centrifugation at 15,800 rpm for 15 minutes at 4°C. Prior to storage, the aliquots were re-aliquoted on a 96-well plate and evaporated to dryness using a TurboVap at 37 °C for 3 to 4 hours.

## Plasma sample reconstitution and quality control

Extracted metabolites were shipped to the Huck Institute of Life Sciences, PA, USA, for ultra-high-performance liquid-chromatography Orbitrap mass-spectrometry. Reconstitution of metabolites was done in HPLC-grade water (Chromasolv, Sigma, St Louis, Missouri) in a 1:1.6 ratio for SPE followed by mixing at 800 rpm for 10 minutes at room temperature and centrifugation on a 96-well plate at 3200 rpm for 15 minutes at 4°C before being transferred to MS microvials (ThermoFisher, Waltham, Massachusetts). Protein precipitated samples were reconstituted in water at a 1:1.4 ratio, mixed and then centrifuged at 15,000 rpm for 15 minutes at 4°C before being transferred to snap-cap low volume MS microvials. An analysis sequence was prepared containing a pooled QC sample and a water blank interspersed between every 10 patient samples. The sequences started with three QC samples and three blanks. Samples were loaded per plate on an auto-sampler and stored at 4°C until analysis. All samples extracted through the same method were run in a single continuous batch with regular inspection for any retention time shifts and changes in mass accuracy among QC samples.

## *P. falciparum* cultures, media sample preparation, and quality control

*P. falciparum* (3D7 strain; MR4, cat. no. MRA-102) was cultured and maintained using standard methods in a mycoplasma-free hood.[10] Culture flasks were kept, with the lid half-closed, in a humidified incubator at 37°C and 5% CO2 and maintained in RPMI-1640 culture medium (Thermo Fisher Scientific) containing 15mmol/L HEPES (Sigma-Aldrich), 2.5g/L Albumax (Thermo Fisher Scientific), 50mg/L gentamycin (Sigma-Aldrich), 2mg/mL sodium-bicarbonate (Sigma Aldrich) and 10mg/L hypoxanthine (Sigma-Aldrich). Cultures were confirmed Mycoplasma-free by PCR using an IntronBio (eMyco) Mycoplasma detection kit (Boca Scientific, No. 25235).

*P. falciparum* cultures were expanded (10% parasitemia) and synchronized twice using 5% sorbitol (Sigma-Aldrich) over two subsequent developmental cycles. The highly synchronized *P. falciparum* culture (in-sync between 4 to 6 hours) was split into three flasks and diluted to 3%, 2%, and 1% parasitemia all kept at 2% hematocrit. Alongside of the parasitized red cell cultures, an uninfected red blood cell control culture was prepared at 2% hematocrit with blood from the same donor. All flasks were given fresh pre-warmed media (37°C) 1 hour before the assays started and were not refreshed thereafter. The culture media were assayed at multiple time points during the intraerythrocytic developmental cycle. Sampling time points corresponded to the parasite stage: small ring stage (6-12 hours post invasion (hpi)), large ring stage (18-24 hpi), trophozoite stage (24-30 hpi), schizont stage (20-36 hpi and 42-48 hpi), re-invasion (48-52 hpi). Spent media were sampled in duplicate at each time point (technical duplicates, n=2), parasitemia was assessed by microscopy and cell counts were done using a hemocytometer.

Metabolite extractions were performed on 100 µL of the spent media by immediately adding 900 µL pre-chilled methanol at (4°C). The methanol extraction solution was spiked with 5 µM of internal standard ^13^C_4_- ^15^N_1_-aspartate to enable post-hoc corrections for variations in sample handling. Precipitated samples were vortexed individually for 30 seconds at full speed followed by centrifugation for 15 minutes at 15,000 rpm and 4°C . A total of 50 µL was transferred to an Eppendorf tube and was evaporated to dryness using nitrogen. In a cold room (4°C), metabolite pellets were re-suspended by adding 67 µL of HPLC-grade water (Chromasolv, Fisher-Scientific) and vortexedfor 30 seconds at full speed, followed by centrifugation for 15 minutes at 15,000 rpm and 4 °C. The final 50 µL of the upper layer was transferred into glass snap-cap low volume MS microvials (ThermoFisher, Waltham, Massachusetts).

For this experiment, fresh RPMI-1640 media (the same as used for the cultures) was used to prepare quality control samples that were regularly interspersed along with water blanks between the media samples. We chose not to use a pooled sample but rather to use fresh RMPI-1640 media which was considered a stable QC and reflected many of the standard constituents that could be found in all media.

## UHPLC-Orbitrap-MS

Metabolites extracted from patient samples and from *Pf*3D7 culture media were analyzed on the same analytical platform and using the same LC-MS parameters. We performed ultra-high-performance liquid-chromatography mass-spectrometry using a Thermo Exactive Plus Orbitrap™ (ThermoFisher, Waltham, Massachusetts). Reversed-phase liquid chromatography was done using a new pre-primed Synergi™ Hydro-RP C18 column (100 x 2mm, particle size 2.5µm, No. 00D-4387-B0, Phenomenex, Torrance, California) fitted with a pre-column filter (Restek, Bellefonte, Pennsylvania). The UHPLC parameters have been described previously (Lu et al. 2010). Data were acquired in negative ion-mode (-ESI) , preceded by calibration using Pierce Negative ESI calibration solution (ThermoFisher, Waltham, Massachusetts). The MS parameters were adapted from previous studies[11] and scanned between m/z 70-1000 with a resolution of 140,000 at m/z 200.

Metabolomics data were converted to .mzXML and optimal peak picking parameters were evaluated using the IPO algorithm (‘Isotopologue Parameter Optimization’) [12]. Mass spectral deconvolution was performed in R (v3.3.3) and MZmine (v2.0)[13]. Metabolites underwent QC-RLSC signal correction and were excluded if they occurred in less than 50% of the QC samples and if the relative standard deviation was $>$20%[14] (Figure S1).

## Amino acid extraction and quantification using HPLC-MS/MS in plasma

Amino acids were extracted from patient plasma samples for amino acid analysis using a Phenomenex EZ:faast™ amino acid analysis kit (Phenomenex, Torrance, California), as described previously.[15] Plasma samples were thawed on ice a sample volume of 100 µL was mixed with 100 µL of a solution containing three internal standards (Homoarginine, Methionine-d3, homophenylalanine). The mixture was then passed through a sorbent tip, and washed with 200 µL N-propanol. Following, the sorbent was eluted by adding a 3:2 mixture of sodium Hydroxide and N-propanol. An organic emulsification was attained by adding chloroform and propyl chloroformate and vortexing the emulsed sorbent for 30 seconds at full speed. Following, iso-octane was added to create an organic solution and the sample was vortexed for 5 seconds at full speed. The upper organic layer containing the extracted metabolites was transferred onto a fresh glass 96 well and evaporated to dryness with nitrogen using TurboVap (Biotage Uppsala, Sweden) for 1 hour at 37°C before the plates were sealed for dry storage at -80°C. Reconstitution of SPE extracted samples was done by adding 100 µL of phase A, into the dry wells and mix for 10 minutes at 600 rpm and room temperature using a MixMate system (ThermoFisher, Waltham, Massachusetts). Reconstituted extracts were transferred into glass snap-cap low volume MS microvials (ThermoFisher, Waltham, Massachusetts) and these were also kept for 1-3 days at -80°C before they were analyzed.

Plasma Free Amino Acids (PFAA) were quantified through LCMS using an ABSciex API 5000 Triple Quadrupole LC/MS/MS Mass Spectrometer. We performed HPLC using an AAA-MS™ column (250 x 3.0 mm, Phenomenex, Torrance, California). Mass spectrometry with multiple reaction monitoring was done in positive ion mode with electro-spray ionization. Prior to data acquisition we optimized the LC system to detect the internal standards. The scan range covered between 100-600 m/z. Inter-assay variation scores were determined for three amino acid standards. Concentrations were based on interpolation on a linear standard curve.

# Supplementary Figures and Tables

**Fig. S1.** Schematic description of metabolic analysis, data normalization and analysis of patient plasma.

**Fig. S2.** Base peak chromatograms of patient plasma extracted using a solid phase extraction (SPE) technique.

**Fig. S3.** Base peak chromatograms of patient plasma extracted using a methanol (MeOH) technique.

**Fig. S4.** Base peak chromatograms of *Pf*3D7 spent media extracted using a methanol protein precipitation (MeOH) technique.

**Fig. S5.** Metabolites detected in a metabolomic analysis (UHPLC-Orbitrap-MS) of plasma collected at study enrolment in study participants (n=152) from Bangladesh. (Part A).

**Fig. S5.** Metabolites detected in a metabolomic analysis (UHPLC-Orbitrap-MS) of plasma collected at study enrolment in study participants (n=152) from Bangladesh. (Part B).

**Figure S6. Metabolomic analysis of patient plasma.** (A) Principal component analysis (PCA) showing separation by patient groups. (B) Differential abundance analysis of acids compared between uncomplicated malaria and healthy controls. (C) Differential abundance analysis of acids compared between patients with uncomplicated malaria and severe malaria. The Y-axis denotes -log10(Benjamini-Hochberg adj. p-values) and x-axis denotes log2(fold change).

**Fig. S7.** Differential abundance analysis of metabolites detected in plasma from patients with uncomplicated malaria (n=47), severe malaria (n=60), and healthy controls (n=45) in a metabolomic analysis (UHPLC-Orbitrap-MS) of plasma. (Part A)

**Fig. S7.** Differential abundance analysis of metabolites detected in plasma from patients with uncomplicated malaria (n=47), severe malaria (n=60), and healthy controls (n=45) in a

metabolomic analysis (UHPLC-Orbitrap-MS) of plasma. (Part B)

**Fig. S8.** Chemical characteristics of suspected microbial acids detected in plasma of acidotic patients with severe P. falciparum malaria. Structure, chemical formula, and average molecular weight (MW) are shown. DAP = diaminopimelic acid. (Chemical data and structures © 2018 HMDB, V4.0).

**Fig. S9.** Temporal kinetics of suspected microbial acids detected in plasma of acidotic patients with severe *P. falciparum* malaria. DAP = diaminopimelic acid.

**Fig. S10.** Correlation plots of *Pf*HRP2 with individual organic acids from a likely enteric bacterial source. *Pf*HRP2 is a marker of parasite biomass and indirectly reflect tissue sequestration.


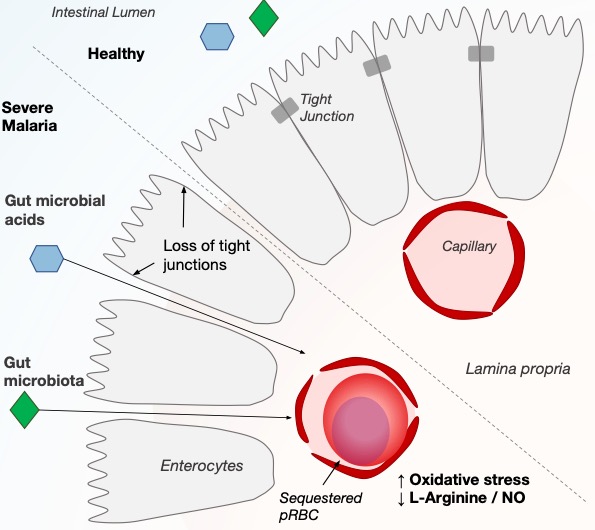


**Figure S11. Proposed mechanism for translocation in severe *P. falciparum* malaria.** Sequestration of parasitized red cells in the splanchnic microcirculation leading to local tissue dysoxia and loss of tight junctions reduces enterocyte integrity, followed by translocation of microbial acids and other bacterial products into the circulation.

**Fig. S12.** Correlation between L-lactate measured using the iStat system and by UHPLC-Orbitrap-MS.

## References

1. Dondorp AM, Desakorn V, Pongtavornpinyo W, et al. Estimation of the total parasite biomass in acute falciparum malaria from plasma PfHRP2. PLoS Med [Internet]. **2005** [cited 2013 Nov 9]; 2(8):0788–0797. Available from: http://www.pubmedcentral.nih.gov/articlerender.fcgi?artid=1188247&tool=pmcentrez&rendertype=abstract

2. Hendriksen ICE, Mwanga-Amumpaire J, Seidlein L von, et al. Diagnosing Severe Falciparum Malaria in Parasitaemic African Children: A Prospective Evaluation of Plasma PfHRP2 Measurement. PLoS Med [Internet]. **2012**; 9(8):e1001297. Available from: http://dx.plos.org/10.1371/journal.pmed.1001297

3. Story DA, Poustie S, Bellomo R. Estimating unmeasured anions in critically ill patients: Anion-gap, base-deficit, and strong-ion-gap. Anaesthesia. **2002**; 57(11):1109–1114.

4. Siggaard-Andersen O. Acid-base balance. Encycl Respir Med. 2005. p. 173–208.

5. Wooten EW. Calculation of physiological acid-base parameters in multicompartment systems with application to human blood. J Appl Physiol [Internet]. **2003**; 95(6):2333–2344. Available from: http://www.physiology.org/doi/10.1152/japplphysiol.00560.2003

6. Stewart PA. Modern quantitative acid-base chemistry. Can J Physiol Pharmacol. **1983**; 61(12):1444–1461.

7. Figge J, Mydosh T, Fencl V. Serum proteins and acid-base equilibria: a follow-up. J Lab Clin Med [Internet]. **1992** [cited 2018 Jan 9]; 120(5):713–719. Available from: http://www.ncbi.nlm.nih.gov/pubmed/1431499

8. Sriboonvorakul N, Leepipatpiboon N, Dondorp AM, et al. Liquid chromatographic-mass spectrometric method for simultaneous determination of small organic acids potentially contributing to acidosis in severe malaria. J Chromatogr B Anal Technol Biomed Life Sci [Internet]. Elsevier B.V.; **2013** [cited 2013 Nov 13]; 941:116–122. Available from: http://www.ncbi.nlm.nih.gov/pubmed/24200840

9. Roberts LD, Souza AL, Gerszten RE, Clish CB. Targeted Metabolomics. Curr Protoc Mol Biol. **2012**; 4(30):1–24.

10. Trager W, Jensen J. Human malaria parasites in continuous culture. Science (80- ) [Internet]. **1976**; 193(4254):673–675. Available from: http://www.sciencemag.org/cgi/doi/10.1126/science.781840

11. Lu W, Clasquin MF, Melamud E, Amador-Noguez D, Caudy AA, Rabinowitz JD. Metabolomic analysis via reversed-phase ion-pairing liquid chromatography coupled to a stand alone orbitrap mass spectrometer. Anal Chem. **2010**; 82(8):3212–3221.

12. Libiseller G, Dvorzak M, Kleb U, et al. IPO: A tool for automated optimization of XCMS parameters. BMC Bioinformatics. **2015**; 16(1):1:10.

13. Pluskal T, Castillo S, Villar-Briones A, Orešič M. MZmine 2: Modular framework for processing, visualizing, and analyzing mass spectrometry-based molecular profile data. BMC Bioinformatics. **2010**; .

14. Dunn WB, Broadhurst D, Begley P, et al. Procedures for large-scale metabolic profiling of serum and plasma using gas chromatography and liquid chromatography coupled to mass spectrometry. Nat Protoc. **2011**; 6(7):1060–1083.

15. Badawy AAB, Morgan CJ, Turner JA. Application of the Phenomenex EZ:faast amino acid analysis kit for rapid gas-chromatographic determination of concentrations of plasma tryptophan and its brain uptake competitors. Amino Acids. **2008**; 34(4):587–596.
